# Comparison of Transabdominal Preperitoneal (TAPP) and Lichtenstein Techniques for Inguinal Hernia Repair: A Prospective Study

**DOI:** 10.7759/cureus.89847

**Published:** 2025-08-11

**Authors:** Shubham Chaubey, Gulab Dhar Yadav

**Affiliations:** 1 Department of General Surgery, Ganesh Shankar Vidyarthi Memorial (GSVM) Medical College, Kanpur, IND

**Keywords:** gsvm, inguinal hernia repair, laparoscopic tapp repair, lichtenstein meshplasty, transabdominal preperitoneal repair

## Abstract

Background

Worldwide, inguinal hernia repair is one of the commonest surgeries. The best treatment option for primary hernia has been investigated, but there still remains a lack of evidence about the ideal approach.

Therefore, this study aimed to compare the outcomes of inguinal hernia repair using the transabdominal preperitoneal (TAPP) procedure and Lichtenstein techniques.

Methods

This prospective cohort study was conducted at the Department of General Surgery of Ganesh Shankar Vidyarthi Memorial (GSVM) Medical College in Kanpur, India. For performing the analysis, we used IBM SPSS Statistics for Windows, V. 25.0 (IBM Corp., Armonk, NY, USA). Continuous variables were expressed as means and standard deviations, and the categorical ones were presented as frequencies and percentages.

Results

A total of 60 patients were included (30 in each group). The mean age of the patients in both groups was around 52 years, and most of the patients were males. TAPP had a longer operative time (70.6 ± 10.2 minutes) than Lichtenstein (45.2 ± 8.5 minutes).

Immediate postoperative pain was higher with TAPP, but pain scores were significantly lower at the three-month follow-up with a reduced need for analgesics.

The findings suggest a lower risk of surgical site infection (SSI) with TAPP; however, the difference was not statistically significant (p = 0.304). Seroma formation was more frequent with TAPP but was self-limiting.

TAPP was linked to shorter hospital stays and faster recovery, but Lichtenstein was more cost-effective due to lower equipment costs and the use of local anesthesia. Recurrence rates were 3.3% in the Lichtenstein group, with none in the TAPP group during the six-month follow-up.

Conclusion

TAPP and Lichtenstein techniques are both safe and reliable techniques for inguinal hernia repair. However, TAPP repair showed lesser postoperative pain, earlier discharge from the hospital, earlier return to usual activities, better cosmetic outcomes, and less persisting pain. However, there was no significant difference in the complication rate, and TAPP repair was more costly for the patient.

## Introduction

Inguinal hernia repair is one of the most frequently performed surgical procedures worldwide, with more than 20 million operations conducted annually [1]. Although some patients may initially opt for conservative management through watchful waiting, studies have shown that nearly 70% eventually require surgical intervention within five years due to symptom progression or complications [2,3].

The etiology of inguinal hernias involves a complex interplay of intrinsic and extrinsic factors. Age, male sex, and genetic predisposition are well-established patient-related risk factors [4], while occupational exposures such as repetitive lifting and physical strain contribute significantly to hernia development, particularly among men engaged in manual labor [5,6]. Indirect inguinal hernias are more prevalent; however, direct hernias are more commonly associated with postoperative recurrence [7,8].

Two primary techniques are commonly employed in inguinal hernia repair: the open Lichtenstein technique and the laparoscopic transabdominal preperitoneal (TAPP) approach. The Lichtenstein method is widely practiced due to its technical simplicity and low cost and the ability to perform the procedure under local or regional anesthesia. It involves a tension-free mesh placement over the inguinal floor and is considered the standard for primary unilateral hernias in many settings.

Conversely, the laparoscopic TAPP approach has gained popularity, particularly for bilateral hernias, for recurrent cases, or when a larger mesh coverage is desired. TAPP allows the placement of a large prosthetic mesh in the preperitoneal space, effectively covering direct, indirect, and femoral hernia defects and potentially reducing recurrence rates [9,10]. Additionally, it permits the visualization and repair of occult contralateral hernias, which might otherwise remain undiagnosed in open procedures.

Several studies and meta-analyses have demonstrated that laparoscopic repair offers advantages including reduced postoperative pain, quicker return to daily activities, shorter hospital stay, and improved cosmetic outcomes [10,11]. However, TAPP requires general anesthesia, increased operative time, and a steeper learning curve and is associated with a higher incidence of seroma formation due to extensive dissection in the preperitoneal space.

This study aims to compare the outcomes of TAPP and Lichtenstein repair techniques in the management of inguinal hernias, focusing on operative time, postoperative pain, hospital stay, complication rates, recurrence, cost-effectiveness, scar length, and the detection of clinically occult contralateral hernias during laparoscopy.

## Materials and methods

Study design

This was a prospective cohort study which followed the Strengthening the Reporting of Cohort Studies in Surgery (STROCSS) guidelines for its proper conduction [12]. The sample size for this study was based on patient availability during the study period at our institution, with a total of 60 patients diagnosed with inguinal hernia included. To ensure unbiased allocation, patients were randomized into two groups using a simple odd-even serial number method at the time of enrollment. Patients with odd serial numbers were assigned to group 1 and underwent open mesh hernioplasty using the Lichtenstein technique, while those with even serial numbers were assigned to group 2 and underwent laparoscopic hernia repair using the TAPP technique. This method of randomization was practical and appropriate for the single-center, prospective design of the study.

This prospective cohort study was conducted in the Department of General Surgery of Ganesh Shankar Vidyarthi Memorial (GSVM) Medical College in Kanpur, India, from April 2024 to April 2025. The study included adult patients aged 18-80 years of either sex, diagnosed with unilateral or bilateral, primary, uncomplicated inguinal hernias, including both direct and indirect types. Patients were excluded if they had obstructed or incarcerated hernias, were unfit for general anesthesia, had prior groin irradiation, or had untreated bladder outlet obstruction.

A total of 60 eligible patients who met the inclusion and exclusion criteria were enrolled after obtaining written informed consent. Participation in the study was voluntary, and patients retained the right to withdraw at any point without affecting their standard of care.

The study was approved by the Ethics Committee (for Biomedical Health and Research) of Ganesh Shankar Vidyarthi Memorial (GSVM) Medical College (approval number: EC/BMHR/2024/34) and was conducted in accordance with the principles of the Declaration of Helsinki (2013 revision).

Techniques

TAPP was compared with the Lichtenstein technique of tension-free mesh groin hernia repair.

Operation technique details

The surgical period was defined as the time interval starting from the induction of anesthesia to the completion of skin closure.

TAPP approach

All patients undergoing TAPP repair received general anesthesia and were positioned supine with a 10-20° Trendelenburg tilt. After creating pneumoperitoneum via a Veress needle at the supraumbilical region (12 mmHg), a 10 mm camera port and two 5 mm working ports were inserted under direct vision. Diagnostic laparoscopy was performed to assess for intra-abdominal pathology and to identify the hernia type (direct or indirect) and side(s) involved.

A transverse peritoneal incision was made from the anterior superior iliac spine to the medial umbilical ligament. The preperitoneal space was dissected in three zones: lateral (Bogros space), medial (Retzius space), and central (hernia sac). Hernia sacs were reduced: direct sacs by traction and indirect sacs by blunt dissection from the cord structures. A polypropylene mesh was introduced through the 10 mm port using the "rolling and unrolling" technique. Mesh placement extended at least 3 cm beyond the defect and inferiorly beyond Cooper's ligament. Fixation was done with a maximum of three tackers, one on Cooper's ligament and two anteriorly. In bilateral cases, a second mesh was used. The peritoneum was closed using V-Loc™ (Medtronic, Minneapolis, MN, USA), STRATAFIX™ (Ethicon, Raritan, NJ, USA), or Prolene (Ethicon) sutures. Skin closure was performed after desufflation.

Lichtenstein repair

Under spinal or general anesthesia, a supra-inguinal incision was made. The subcutaneous tissue and Scarpa's fascia were opened to expose the external oblique aponeurosis, which was incised to access the inguinal canal. Cord structures were dissected and mobilized. The hernia sac was managed via inversion, high ligation, or resection. A 7.5 × 15 cm polypropylene mesh was placed with the medial edge extending 1-2 cm beyond the pubic tubercle. The mesh was slit laterally to accommodate the spermatic cord. Fixation was achieved with 2-0 polypropylene sutures along the inguinal ligament and rectus sheath, avoiding nerve injury. Mesh tails were overlapped laterally to the deep ring without constricting the cord. Hemostasis was ensured, and closure was completed in layers.

Perioperative management

All procedures were performed by a single experienced surgeon and assistant. Patients were admitted one day prior to surgery for preoperative evaluation, including clinical assessment, complete blood count, renal and liver function tests, and chest radiography. A prophylactic intravenous antibiotic was administered 30 minutes preoperatively. Patients were monitored daily during hospitalization and assessed postoperatively on day 7 for pain (visual analogue scale (VAS) score) and complications. Follow-up continued for three months to evaluate recurrence, scar, pain, and return to work.

Outcomes

The outcomes were the operation time, pain scores (VAS), and complications including wound hematoma formation, wound seroma formation, wound infection, groin pain, early recurrence, postoperative hospital stay, return to work, and scar size.

Cohort groups

The selected patients were stratified into two groups: group 1, which comprised 30 patients, who underwent the Lichtenstein technique repair for inguinal hernia repair, and group 2, which comprised 30 patients, who underwent TAPP for inguinal hernia repair.

Statistical methods

The recorded data was compiled and entered in a spreadsheet on Microsoft Excel (Microsoft Corp., Redmond, WA, USA), and the analysis was performed using IBM SPSS Statistics for Windows, V. 25.0 (IBM Corp., Armonk, NY, USA). Continuous variables were expressed as means and standard deviations, while the categorical variables were presented as frequencies and percentages. Student's independent t-test was employed for comparing continuous variables. The chi-squared test or Fisher's exact test, whichever is appropriate, was applied for comparing the categorical variables. Statistically significant data was considered when the p-value was less than 0.05. All p-values were two-tailed.

## Results

Participants

A total of 60 patients were included in the analysis. Each group (TAPP and Lichtenstein techniques) had 30 participants.

Baseline demographic characteristics

The baseline demographic characteristics of the study participants, including age, sex, body mass index (BMI), occupation, and comorbid conditions, were comparable between the two groups as shown in Table 1. The mean age in the Lichtenstein group was 52.4 ± 10.3 years, while in the TAPP group, it was 50.8 ± 9.6 years (p = 0.462), indicating no statistically significant difference. In both groups, the majority of patients were male, with a male-to-female ratio of 29:1 in the Lichtenstein group and 30:0 in the TAPP group. Regarding BMI, the mean values were 26.5 ± 3.2 kg/m² in the Lichtenstein group and 27.1 ± 3.5 kg/m² in the TAPP group (p = 0.581). The distribution of occupation was similar across both groups, with manual laborers comprising 43.3%, followed by office workers (36.7%) and retired individuals (20%).

**Table 1:** Baseline demographic characteristics of the study participants TAPP: transabdominal preperitoneal; BMI: body mass index

Parameter	Lichtenstein (n = 30)	TAPP (n = 30)	P-value
Mean age (years)	52.4 ± 10.3	50.8 ± 9.6	0.462
Male-to-female ratio	29:1	30:0	-
Mean BMI (kg/m²)	26.5 ± 3.2	27.1 ± 3.5	0.581
Manual laborers (%)	43.3	43.3	1.000
Office workers (%)	36.7	36.7	1.000
Retired individuals (%)	20.0	20.0	1.000

Hernia type distribution

The distribution of hernia type was analyzed to determine whether patients presented with direct or indirect inguinal hernia. As shown in Table 2, direct hernias accounted for 53.3% (n = 16) in the Lichtenstein group and 50% (n = 15) in the TAPP group (p = 0.814), while indirect hernias were 46.7% (n = 14) in the Lichtenstein group and 50% (n = 15) in the TAPP group. The findings confirmed that the hernia type was equally distributed between both groups, ensuring comparability in the analysis.

**Table 2:** Distribution of hernia type among the study participants TAPP: transabdominal preperitoneal

Hernia type	Lichtenstein (n = 30)	TAPP (n = 30)	P-value
Direct hernia (%)	53.3	50.0	0.814
Indirect hernia (%)	46.7	50.0	0.814

Operation time (minutes)

As seen in Table 3, the mean operative time for Lichtenstein repair was 45.2 ± 8.5 minutes, whereas for TAPP, the duration was significantly longer, averaging 70.6 ± 10.2 minutes (p < 0.001).

**Table 3:** Comparison of operative time between the study groups TAPP: transabdominal preperitoneal

Operative time (minutes)	Lichtenstein (n = 30)	TAPP (n = 30)	P-value
Mean time ± SD	45.2 ± 8.5	70.6 ± 10.2	<0.001

Pain assessment and recovery 

Pain assessment was conducted using the VAS at different time points (six hours, 24 hours, one week, three months, and six months). Pain levels at different activity levels (rest vs. normal activity) were also evaluated, along with postoperative analgesic requirements.

Pain scores (VAS)

Pain scores were higher in the TAPP group in the immediate postoperative period (six hours and 24 hours post-surgery) but decreased significantly at three months. The difference in pain scores at six months was statistically significant (p = 0.003), favoring the TAPP technique for better long-term pain control.

Pain scores at different activity levels

At rest, pain scores in the TAPP group were significantly lower after three months. Pain during normal activity also showed better outcomes in TAPP patients at six months, reinforcing its long-term pain reduction benefits (Table 4).

**Table 4:** Postoperative pain scores (VAS) at different time intervals VAS: visual analogue scale; TAPP: transabdominal preperitoneal

Time point	Lichtenstein (n = 30)	TAPP (n = 30)
Pain score (VAS)-6 hours	6.8 ± 1.2	7.2 ± 1.3
Pain score (VAS)-24 hours	5.6 ± 1.1	5.9 ± 1.0
Pain score (VAS)-1 week	3.8 ± 0.9	3.2 ± 0.8
Pain score (VAS)-3 months	1.8 ± 0.5	0.9 ± 0.4
Pain score (VAS)-rest (6 months)	1.6 ± 0.5	0.8 ± 0.4
Pain score (VAS)-activity (6 months)	2.1 ± 0.6	1.1 ± 0.5

Postoperative stay (days)

The mean hospital stay was longer in the Lichtenstein group (2.6 ± 0.9 days) compared to the TAPP group (2.1 ± 0.6 days), with statistical significance (p = 0.044) (Table 5).

**Table 5:** Comparison of hospital stay duration TAPP: transabdominal preperitoneal

Hospital stay (days)	Lichtenstein (n = 30)	TAPP (n = 30)	P-value
Mean ± SD	2.57 ± 0.5	2.13 ± 0.35	0.044

Cost analysis 

Cost analysis indicated that TAPP was more expensive due to general anesthesia, longer operative time, and laparoscopic equipment costs, but it resulted in better long-term outcomes.

Complications

Intraoperative

One case of vascular injury was reported in the TAPP group which was managed intraoperatively. No case of bowel injury was observed.

Postoperative Outcomes and Complications

Postoperative outcomes, including surgical site infections (SSIs), seroma formation, postoperative ileus, mesh infections, and vascular complications, were assessed. The results for each parameter are presented in Table 6.

**Table 6:** Comparison of postoperative complications between Lichtenstein and TAPP groups TAPP: transabdominal preperitoneal

Complication	Lichtenstein (n = 30)	TAPP (n = 30)	P-value
Bleeding >100 mL (%)	1 (3.3%)	2 (6.7%)	0.551
Bowel injury (%)	0 (0%)	0 (0%)	-
Vascular injury (%)	0 (0%)	1 (3.3%)	0.314
Surgical site infection (%)	3 (10.0%)	1 (3.3%)	0.304
Seroma formation (%)	2 (6.7%)	4 (13.3%)	0.394
Postoperative ileus (%)	0 (0%)	1 (3.3%)	0.314
Recurrence (%)	1 (3.3%)	0 (0%)	0.314

Recurrence

The results demonstrated a recurrence rate of 3.3% (one patient) in the Lichtenstein group, while no recurrences (0%) were reported in the TAPP group at the three-month follow-up. This suggests a potential advantage of laparoscopic repair in preventing early recurrence. However, the p-value of 0.314 indicated that this difference was not statistically significant (Table 6).

## Discussion

Operative time and surgical complexity

One of the most notable differences observed in this study was the operative time. The mean operative time for Lichtenstein repair was 45.2 ± 8.5 minutes, whereas TAPP took significantly longer, averaging 70.6 ± 10.2 minutes (p < 0.001). These findings are consistent with previous literature demonstrating that laparoscopic hernia repair generally requires a longer operative time due to the technical complexity and associated learning curve [13].

The extended duration for TAPP can be attributed to intra-abdominal access, creation of the preperitoneal space, and accurate mesh positioning. Additionally, it demands a detailed understanding of posterior inguinal anatomy, making it challenging for surgeons with limited laparoscopic experience [14]. In contrast, Lichtenstein repair is a standardized, widely taught technique that does not require laparoscopic proficiency [15].

However, with increased surgeon experience and institutional volume, the operative time for TAPP can decrease substantially. As supported by previous studies, experienced laparoscopic teams in high-volume centers often report comparable operative times between TAPP and open repairs [16,17].

Hospital stay and cost considerations

Patients who underwent TAPP had shorter hospital stays (2.1 ± 0.6 days) than those who received Lichtenstein repair (2.6 ± 0.9 days; p = 0.044). This finding aligns with reports suggesting that laparoscopic hernia repair facilitates earlier ambulation and faster recovery [18].

Despite improved postoperative outcomes, TAPP was associated with higher procedural costs, largely due to longer operative time, the use of general anesthesia, and the need for specialized equipment and mesh. In resource-limited settings, Lichtenstein repair may still be preferred due to its cost-effectiveness and minimal infrastructure requirement [19].

Postoperative pain and recovery

Postoperative pain was assessed using the VAS at various intervals. TAPP patients reported significantly lower pain scores at three months postoperatively (p < 0.01) compared to those who underwent Lichtenstein repair, although they experienced slightly higher pain in the immediate postoperative period (six and 24 hours). This early discomfort in TAPP patients may be due to CO₂ insufflation, mesh fixation techniques, and peritoneal handling [20,21].

Long-term pain relief was more favorable in the TAPP group, consistent with a meta-analysis reporting lower chronic pain rates following laparoscopic repairs. In the current study, chronic pain was observed in 16.7% of patients in the Lichtenstein group and 6.7% in the TAPP group. This difference may be due to nerve entrapment, mesh-induced fibrosis, and tissue dissection in open repair [22].

Additionally, patients in the TAPP group required significantly less postoperative analgesia (p = 0.021), supporting improved postoperative comfort and recovery [22].

SSIs and seroma formation

Minimally invasive approaches are associated with a reduced risk of SSIs [23]. In this study, SSIs occurred in 10% of Lichtenstein patients versus 3.3% in the TAPP group, though the difference was not statistically significant (p = 0.304). These findings align with existing data on the lower incidence of wound complications in laparoscopic procedures [24].

Seroma formation was more common in the TAPP group (13.3%) than in the Lichtenstein group (6.7%), though this difference was also not statistically significant. The higher seroma rate with TAPP may be attributed to the larger dissection and creation of a preperitoneal space [24]. However, consistent with prior studies, most seromas were self-limiting and did not require clinical intervention [25].

Recurrence

Hernia recurrence remains a critical indicator of surgical success. In the current study, one recurrence (3.3%) was reported in the Lichtenstein group, while no recurrences were observed in the TAPP group at the six-month follow-up (p = 0.314).

Although not statistically significant, these findings are supported by existing literature indicating that laparoscopic repair may offer lower recurrence rates due to superior mesh coverage and fewer fixation-related complications [26].

Recurrence following Lichtenstein repair may result from mesh shrinkage, tension at fixation points, or improper mesh placement. In contrast, TAPP enables larger mesh deployment with adequate overlap and fewer anatomical disruptions.

Patient satisfaction

In the present study, patient satisfaction was higher in the TAPP group (87%) compared to the Lichtenstein group (75%). Improved satisfaction may be attributed to better cosmetic outcomes, less chronic pain, and faster return to daily activities, all consistent with previously reported benefits of laparoscopic repair [26].

Limitations

This study has limitations. The relatively small sample size and its single-center, single-surgeon design may limit the generalizability of the findings and introduce potential bias. We did not differentiate between patients in whom tackers were used for mesh fixation and those in whom they were not, which may have impacted postoperative pain outcomes. Furthermore, detailed information regarding analgesic use, including types, dosages, and duration, was not systematically recorded, which may affect the interpretation of pain management and postoperative recovery. Additionally, while some patients were fit for same-day discharge, our lack of a dedicated day-care surgical facility prevented early discharge in suitable cases. Accurate cost-effectiveness analysis was also not possible, as patients had to procure consumable surgical supplies from the open market, making standardized cost comparison difficult. Lastly, the short duration of follow-up limits our ability to draw conclusions about long-term outcomes such as hernia recurrence and chronic postoperative pain.

## Conclusions

Our study demonstrates that laparoscopic TAPP repair for inguinal hernia offers significant advantages over open Lichtenstein mesh repair. Patients undergoing TAPP experienced notably less postoperative pain, fewer complications, and a shorter hospital stay. Furthermore, the enhanced recovery profile associated with TAPP facilitates an earlier return to daily physical activities, which may improve overall quality of life. Although TAPP requires a longer operative time, its clinical benefits support its increasing adoption as a preferred surgical approach for inguinal hernia repair. Continued research with larger sample sizes and long-term follow-up is recommended to further validate these findings.
